# Chromosomal architecture changes upon cell differentiation

**DOI:** 10.1186/1756-8935-6-S1-P130

**Published:** 2013-04-08

**Authors:** Maxim Imakaev, Geoffrey Fudenberg, Leonid Mirny

**Affiliations:** 1 Department of Physics, Massachusetts Institute of Technology, Cambridge, MA, USA; 2 Harvard University, Program! in Biophysics, Boston, Massachusetts, USA; 3 Institute for Medical Engineering and Science, MIT, USA

## Background

The recently developed Hi-C method provides a comprehensive whole-genome picture of physical contacts between distal loci. Analysis of these data has begun to reveal determinants of 3D genomic organization. However, the similarities and differences in chromosomal organization between cell-types remain unexplored.

## Materials and methods

To analyze chromosomal architecture between cell types, it is crucial to have a consistent way of analyzing Hi-C data and removal of experimental biases. To this end we developed a comprehensive method of Iterative Correction and Eigenvector decomposition (ICE)^2^. ICE maps Hi-C reads to the genome, filters mapped reads and obtains a Hi-C map of relative contact probabilities free of experimental biases. It then decomposes the maps into a set of genomic tracks characterizing high-order chromatin organization.

## Results

Using ICE, we analyze Hi-C data^3^ from human embryonic stem cells, and IMR90 lung fibroblast cells. We focus our analysis on the compartment profile, which has been shown to partition the genome into transcribed gene-rich regions, enriched in active chromatin marks (“active” regions), and “inactive” gene-poor regions. First, we show that ES cells have a gradual transition between “active” and “inactive” chromatin interaction preferences, as demonstrated by a broad unimodal distribution of values of the compartment profile. In contrast, differentiated IMR90 cells show one inactive chromatin state and a range of states at the active end. Second, we find that chromatin interactions in embryonic cells are best described by GC content of a genomic region. Conversely, for the differentiated cell line IMR90, transcription data (CAGE) is a much better predictor of chromatin interaction preferences than sequence-derived features. Lastly, we analyze changes in chromatin interactions upon differentiation, and find that regions which belonged to an active compartment in ES cells often switch to inactive compartment in IMR90, while the opposite rarely happens.

## Conclusions

Taken together, our results show that genome-wide chromatin interactions change upon differentiation of ES cells into IMR90, and suggest that sequence-dependent chromatin interactions in embryonic stem cells get overridden in a cell-type-specific manner. We show that upon differentiation regions change from an active to an inactive compartment, suggesting that change in chromatin interactions reflects cell-type-specific silencing of genomic regions.

## References

[B1] Lieberman-Aiden E van Berkum NL Williams L Imakaev M Ragoczy T Telling A Dekker J Comprehensive mapping of long-range interactions reveals folding principles of the human genome Science 2009 326 5950 289 293 10.1126/science.1181369 19815776 PMC2858594

[B2] Imakaev M Fudenberg G McCord RP Naumova N Goloborodko A Lajoie BR Mirny LA Iterative correction of Hi-C data reveals hallmarks of chromosome organization Nature Methods 2012 9 10 999 1003 10.1038/nmeth.2148 22941365 PMC3816492

[B3] Dixon JR Selvaraj S Yue F Kim A Li Y Shen Y Ren B Topological domains in mammalian genomes identified by analysis of chromatin interactions Nature 2012 485 7398 376 338 10.1038/nature11082 22495300 PMC3356448

